# The role of PET/CT in cervical cancer

**DOI:** 10.3389/fonc.2013.00034

**Published:** 2013-02-26

**Authors:** Fernanda G. Herrera, John O. Prior

**Affiliations:** ^1^Department of Radiation Oncology, Lausanne University HospitalLausanne, Switzerland; ^2^Nuclear Medicine, Lausanne University HospitalLausanne, Switzerland

**Keywords:** cervical cancer, positron emission tomography, Fluorodeoxyglucose F18, radiation therapy, Planning Treatment Volumes

## Abstract

In locally advanced cervical cancer, ^18^F-fluorodeoxyglucose (FDG) positron emission tomography – computed tomography (PET/CT) has become important in the initial evaluation of disease extent. It is superior to other imaging modalities for lymph node status and distant metastasis. PET-defined cervical tumor volume predicts progression-free and overall survival. Higher FDG uptake in both primary and regional lymph nodes is strongly predictive of worse outcome. FDG-PET is useful for assessing treatment response 3 months after completing concurrent chemo-radiotherapy (CRT) and predicting long-term survival, and in suspected disease recurrence. In the era of image-guided adaptive radiotherapy, accurately defining disease areas is critical to avoid irradiating normal tissue. Based on additional information provided by FDG-PET, radiation treatment volumes can be modified and higher doses to FDG-positive lymph nodes safely delivered. FDG-PET/CT has been used for image-guided brachytherapy of FDG-avid tumor volume, while respecting low doses to bladder and rectum. Despite survival improvements due to CRT in cervical cancer, disease recurrences continue to be a major problem. Biological rationale exists for combining novel non-cytotoxic agents with CRT, and drugs targeting specific molecular pathways are under clinical development. The integration of these targeted therapies in clinical trials, and the need for accurate predictors of radio-curability is essential. New molecular imaging tracers may help identifying more aggressive tumors. ^64^Cu-labeled diacetyl-di(N(4)-methylthiosemicarbazone) is taken up by hypoxic tissues, which may be valuable for prognostication and radiation treatment planning. PET/CT imaging with novel radiopharmaceuticals could further impact cervical cancer treatment as surrogate markers of drug activity at the tumor microenvironment level. The present article reviews the current and emerging role of PET/CT in the management of cervical cancer.

## INTRODUCTION

Cervical cancer is the second most common cancer among women in the world, and a leading cause of cancer mortality, affecting mainly the under deserved populations of sub-Saharan Africa, Central and Latin America, and South-Central Asia (Ferlay et al., 2010a,b). Clinical staging of cervical cancer is based on the International Federation of Gynaecology and Obstetrics (FIGO) system, which was revised in 2009 (Pecorelli et al., 2009). This staging system is based on physical examination and inspection with scarce radiographic evaluation, aiming to be easily introduced in non-developed nations with limited access to imaging studies. However, compared with surgical staging, clinical examinations alone can under-stage cervical cancer in 20–30% of stage IB and up to 64% of stage IIIB patients (Lagasse et al., 1980). Improvements in tumor staging by imaging modalities, such as computed tomography (CT), magnetic resonance imaging (MRI), and fluorine-18-labeled fluoro-2-deoxy-D-glucose positron emission tomography (FDG-PET) can significantly improve treatment decisions and the accuracy of highly precise radiotherapy.

Locally advanced cervical cancer is treated with chemo-radiotherapy (CRT), which has shown to improve local control and survival. Nevertheless, increasingly more radio- and chemo-resistant tumors still recur. New research strategies have focused on the development of tumor biomarkers aiming to combine CRT with new molecular targets. In this setting FDG-PET/CT and other molecular tracers might help to identify more aggressive tumors.

The aim of this article is to review the evidence and illustrate the role of FDG-PET/CT in the pre-treatment evaluation, disease delineation, and treatment response, with a particular focus on new and emerging metabolic tracers that could eventually performed better as biomarkers of tumor response to therapy.

## STAGING OF CERVICAL CANCER

### PRIMARY TUMOR

The local extent of cervical carcinoma is usually determined by clinical examination, often performed under anesthesia. Considering imaging modalities, MRI has been shown to be the best examination due to its soft tissue resolution and multiplanar capabilities, allowing the accurate determination of tumor volume, size, and parametrial infiltration. The range of accuracy of MRI is 90–100%, as compared with 60–70% for CT. MRI is considered the gold standard method to evaluate loco-regional extension of cervical cancer.

Fluorine-18-labeled fluoro-2-deoxy-D-glucose positron emission tomography – computed tomography can also be used in the initial evaluation of the primary tumor, which is usually FDG-avid, and can provide additional information regarding involved lymph nodes, and distant metastases.

Wong et al. (2004) reported a series of 61 patients with cervical cancer who had a FDG-PET in the initial work-up. Their conclusion was that the PET was able to detect 100% of primary cervical tumors. Another study, which included 60 patients, and reported low sensitivity of FDG-PET for patients with cervical cancer stage 1A2–2A, but this study was performed without combined CT (Chou et al., 2006). It has been demonstrated that PET/CT has a higher accuracy than separate PET and CT scans read side-by-side (Metser et al., 2005).

The degree of FDG-activity in the primary tumor, as measured by the maximum standardized uptake value (SUV_max_), is a predictive biomarker of lymph node status and disease outcome (Kidd et al., 2007). Cervical cancer histology and tumor differentiation has shown to affect FDG uptake. In a study performed by Kidd et al. (2009), 240 women with cervical cancer stage IB2–IVB were evaluated with pre-treatment FDG-PET/CT. In this study the mean SUV_max_ was significantly different between well differentiated vs. poorly differentiated tumors (*p* = 0.047). Squamous vs. non-squamous tumors demonstrated a significant difference in SUV_max_ (*p* = 0.015). The influence of tumor volume as a prognostic factor in cervical cancer has been previously established (Eifel et al., 1994; Fyles et al., 1995; Perez et al., 1998). Poor regression of initial tumor volume has been found by several groups to confer a poor overall survival. Mayr et al. (2002) used MRI scans to evaluate tumor regression at 40–50 Gy of external beam RT combined with chemotherapy in 34 cervical cancer patients. Regression to less than 20% of residual tumor volume resulted in a cumulative incidence of local recurrence of 9.5 vs. 77% in patients with more than 20% residual volume (*p* < 0.001).

In line with these results, a recent prospective study that included 32 patients who underwent FDG-PET/CT during the course of radiotherapy showed that after 19.8 Gy of external beam radiotherapy, the mean physiologic tumor volume was reduced from 102 to 72 cm^3^, representing a 29% reduction in volume (Lin et al., 2006). After an additional 13 Gy from high dose rate (HDR) brachytherapy, the mean volume was reduced to 15.4 cm^3^ and subsequently to 8.6 cm^3^. Patients with residual disease after 3 months of CRT had a worst outcome. This study has important implications for the use of image-guided adaptive radiotherapy. For example, patients with important tumor response during the course of treatment can potentially benefit from dose-volume modifications, which might help to reduce acute and late toxicity, whilst patients with persistent disease might be candidates for other research strategies such as adjuvant chemotherapy or evaluation of new biological therapy (Gaffney, 2005; Herrera et al., 2006; Duenas-Gonzalez et al., 2011; Townsley et al., 2011; Gaffney et al., 2012; Schefter et al., 2012).

**FIGURE 1:**
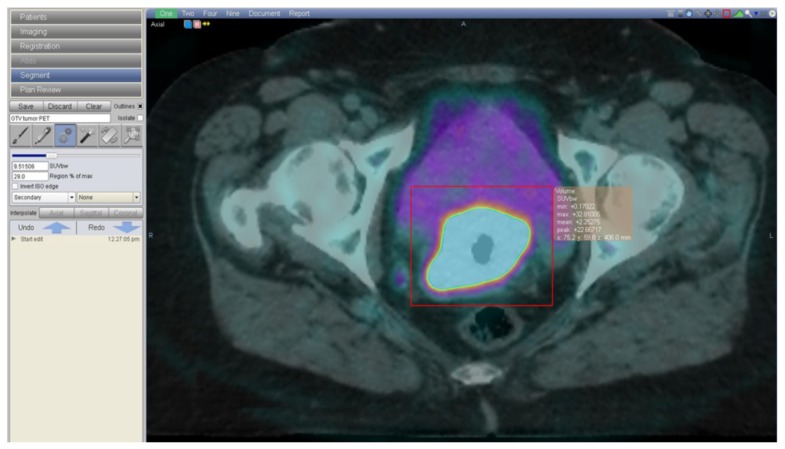
**A 62-year-old woman with a FIGO IIB cervical cancer, treated with concomitant cisplatin based chemotherapy and radiotherapy**. Image shows the radiotherapy contouring process on fused planning-CT and FDG-PET/CT images. Contouring is done on VelocityAI Software (Velocity, Atlanta, GA, USA), based on a method of automatic 3D volume segmentation of the functional image, that depends on the relationship between source to background ratio.

A recent publication evaluating 47 patients with stage IB–IV cervical cancer compared quantitative and qualitative discrepancies between MRI and PET/CT using a conformity index and an overlap factor (Ma et al., 2011). Tumor volume measurements were not statistically different with either modality, although the study shows that for tumors larger than 60 cm^3^ the overlap factor was 0.68, indicating 32% discordance, and for smaller tumors the overlap factor fell to 0.28, indicating 72% discordance. The authors concluded that MRI and PET/CT show a similar performance in evaluating tumor volume but that the location of the tumor can vary significantly between these two imaging modalities possibly due to tumor and organ movement between scans. This has important implications for contouring the gross tumor volume (GTV) in radiotherapy. In our institution, both imaging modalities are fused on the planning-CT. To delineate tumor GTV on fused PET/CT-planning CT, we use a method of automatic 3D volume segmentation of the functional image based on the relationship between source to background ratio. The lesion is segmented based on a given level of radio-activity from the functional image (Daisne et al., 2003). In our clinic, we have chosen Velocity Advanced Image Software (Atlanta, USA), a commercially available software, which provides a different algorithm to auto-segment the region of interest based on the principles previously described (**Figure 1**). Both MRI-GTV and automated segmented FDG-PET/CT-GTV are then joining for accurate delineation of the final GTV.

### NODAL STAGING

**FIGURE 2:**
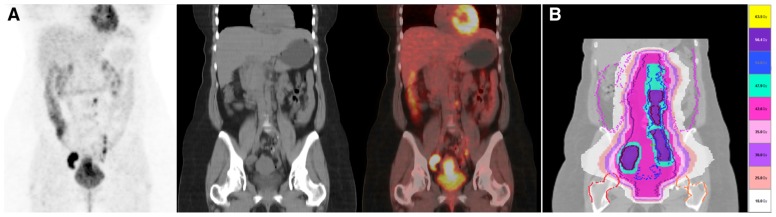
**(A)** A 48 year-old lady with a cervical cancer stage FIGO IIB, presenting with multiple positive lymph nodes in continuity located in the bilateral iliac and para-aortic regions on FDG PET/CT. She was treated with chemo-radiotherapy using helical TomoTherapy. **(B)** Three level of radiotherapy dose were design and treated simultaneously. Pelvis and para-aortic areas received 44.8 Gy/1.6 Gy in 28 fractions. The PAO and pelvis regions surrounding positive nodes but without metabolic uptake were treated with 50.4 Gy/1.8 Gy in 28 fractions. Positive FDG PET/CT lymph nodes were treated with a simultaneous integrated boost up to 59.36 Gy/2.12 Gy in 28 fractions. Scale dose banding shows the 95% of the dose.

Nodal status can significantly influence disease outcome with 90% overall survival in patients with small tumors and negative lymph nodes, and less than 50% in patients with positive pelvic lymph nodes. Patients with positive para-aortic lymph nodes have a bleak prognosis with an overall survival of <20–30% at 5 years. The evaluation of nodal status can therefore have a tremendous impact in the treatment planning with radiotherapy. For example, the presence of metastatic lymph nodes in the pelvis or para-aortic area can lead to plan an intensity-modulated radiation therapy (IMRT)-integrated boost with dose escalation on that involved area (Kidd et al., 2010b; **Figures 2A**,**B**). Tsai et al. (2004) found that 28% of patients had their treatment modified due to additional PET findings in untreated cervical cancer with MRI-defined pelvic node metastasis.

Positron emission tomography – computed tomography is more accurate than CT for evaluating lymph node staging, although the sensitivity and specificity of FDG-PET/CT are variable depending on the stage of the disease (Kidd et al., 2010a). In early stage disease PET/CT has a sensitivity of 53–73%, and a specificity of 90–97% for the detection of lymph node involvement (Reinhardt et al., 2001; Roh et al., 2005; Wright et al., 2005; Chou et al., 2006; Sironi et al., 2006).

In more advance disease (>IB2), the sensitivity for detecting para-aortic lymph node involvement increases to 75% with a specificity of 95%. PET sensitivity has been reported to be superior to MRI. Sugawara et al. (1999) reported 86% FDG-PET sensitivity for pelvic and para-aortic lymph node metastasis, compared with a CT sensitivity of 57% in a series of 21 patients with advanced cervical cancer. Rose et al. (1999) reported a study of locally advanced cervical cancer assessed by PET before surgical staging, in which FDG-PET had a sensitivity of 75%, a specificity of 92%, a positive predictive value (PPV) of 75% and a negative predictive value (NPV) of 92% for para-aortic lymph node metastasis.

In the series of the Gustave Roussy Institute, histological results of complete para-aortic lymphadenectomy were reported in patients treated for stage IB2/II cervical carcinoma who had no para-aortic uptake on FDG-PET/CT: three out of thirty-eight patients had histologically proven para-aortic involvement (metastatic nodes with capsular rupture in the para-aortic area), leading to a NPV of 92% for para-aortic nodal involvement (Boughanim et al., 2008).

Grigsby et al. (2001) retrospectively studied 101 patients before primary CRT. CT scan demonstrated abnormal pelvic lymph nodes in 20% and para-aortic lymph nodes in 7%, while PET/CT detected abnormal FDG uptake in the pelvic lymph nodes in 67%, in the para-aortic lymph nodes in 21% and in the supraclavicular lymph nodes in 8%. The 2-year progression-free rates were 64% for CT (-) PET (-); 18% for CT (-) PET (**+**); and 14% for CT (**+**) PET (**+**) (*p* < 0.0001). A recent up-date of that study which finally enrolled 560 patients treated with surgery alone, surgery and post-operative radiotherapy, or definitive CRT, showed that in 47% of patients, lymph node involvement had been shown on FDG-PET/CT at diagnosis (Kidd et al., 2010a). The frequency of lymph node metastasis was similar to that in historical surgical series and increased according to the clinical stage. Patients with PET-positive lymph nodes had significantly worse disease-specific survival than those with PET-negative lymph nodes (*p* < 0.001). Disease-specific survival was stratified into distinct groups based on the most distant level of PET-detected nodal disease (none, pelvic, para-aortic, or supraclavicular). The hazard ratios for disease recurrence increased incrementally based on the most distant level of nodal disease: pelvic 2.4 (95% CI, 1.6–3.5), para-aortic 5.9 (95% CI, 3.8–9.1), and supraclavicular 30 (95% CI 17–55).

Most significantly, in a subgroup of 83 patients with positive FDG-PET/CT lymph nodes, the lymph node SUV_max_ was predictive of treatment response, risk of pelvic disease recurrence, disease-specific survival, and overall survival. The SUV_max_ at the level of the lymph nodes was found to be predictive of persistent disease in the pelvic lymph node region after treatment, and more than 80% of patients who demonstrated persistent disease in their post-treatment FDG-PET/CT were eventually confirmed to have a pelvic disease recurrence (Kidd et al., 2010b).

These results have important implications for treatment decisions, and raise the question if lymphadenectomy staging is still necessary. Narayan et al. (2001) compared PET with MRI and assessed whether using either of these methods would avoid surgical staging in 27 patients with locally advanced cervical carcinoma assigned to receive local radiotherapy. PET demonstrated sensitivity superior to MRI, and had a PPV of 98% to detect para-aortic lymph node metastasis. However, small volume micro-metastatic disease was still missed on PET. They recommended para-aortic lymphadenectomy in all patients with positive pelvic nodes on PET.

In our institution, independently of the FDG-PET status, we routinely perform lymphadenectomy as a standard approach. This has the advantage of detecting the 5–8% positive lymph nodes not visible on PET allowing a better treatment assignment of either surgery or CRT for early stage disease.

## EVALUATION OF TREATMENT RESPONSE AND DISEASE RECURRENCE

One third of patients with locally advanced cervical cancer will have disease recurrence, usually within 2 years of completing treatment. Predictors of disease recurrence include clinical stage, lymph node status at diagnosis, and tumor response after treatment.

After CRT as definitive treatment of locally advanced cervical cancer there is sufficient evidence to support the use of PET/CT for the assessment of treatment response. The presence of FDG activity (either persistent or new) can predict survival outcome. A study in which FDG-PET/CT was performed 3 months after completion of treatment showed that a metabolic response was predictive of long-term survival, with a 3-year survival rate of 78% in patients with a complete metabolic response, 33% in patients with a partial metabolic response, and 0% in those with progressive disease (Schwarz et al., 2007). Multivariate analysis in that study showed that post-treatment response and lymph node status at diagnosis were the only accurate predictors of progression-free survival.

Mayr et al. (2002) used MRI scans to evaluate tumor regression at 40–50 Gy of external beam RT combined with chemotherapy in 34 cervical cancer patients. Regression to less than 20% of residual tumor volume resulted in a cumulative incidence of local recurrence of 9.5 vs. 77% in patients with more than 20% residual volume (*p* < 0.001).

**FIGURE 3:**
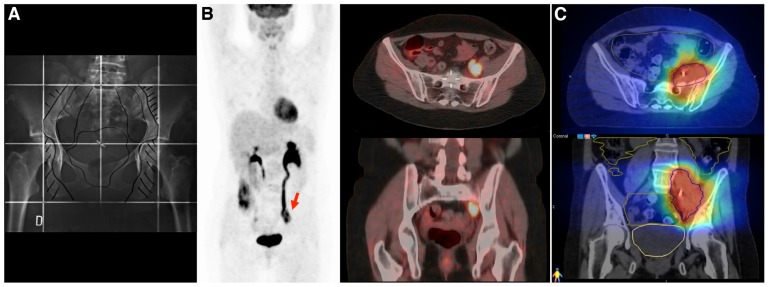
**A 43 year-old women with cervical cancer, FIGO IIB, without Kindly specify the same**. evidence of macroscopic positive nodes at diagnosis. She was treated with chemotherapy and 3D conformal radiotherapy (45 Gy/1.8 Gy/fraction) followed by brachytherapy. **(A)** The initial radiotherapy field does not include the irradiation of common iliac nodes. **(B)** An FDG-PET/CT performed 2 years after primary treatment shows an isolated left iliac recurrence (arrow). This recurrence is observed near the border of the radiation field, which in the context of centrally controlled cervical cancer makes us suspect a component of marginal recurrence that typically arise immediately adjacent to the radiotherapy border. Surgical intervention was considered unfeasible and she underwent salvaged chemotherapy (carboplatin and taxane), followed by re-irradiation. **(C)** Re-irradiation was performed with helical tomotherapy using a hypofractionated schema of 15 daily fractions of 3.5 Gy. All tomotherapy plans were processed on VelocityAI to evaluate cumulative dose to normal tissue and organs at risk (OAR). Megavoltage computed tomography (MVCT) was performed every day before treatment to correct patient setup. The patient is alive without evidence of disease at the 3-year follow-up.

Standardized surveillance programs have proposed the use of routine physical examination and patient’s symptoms education to facilitate early disease detection. However, studies have reported better overall survival in patients with asymptomatic disease recurrence (Bodurka-Bevers et al., 2000). In that setting, the use of FDG-PET/CT in a selected group of patients could potentially lead to a salvage curative therapy of local or oligometastatic disease (Brooks et al., 2009). In a study performed by Mittra et al. (2009), 30 women with locally advanced tumors who had undergone FDG-PET/CT during the surveillance period were evaluated. FDG-PET/CT facilitated the detection of local and distant metastasis, with a sensitivity of 93–96% and a specificity of 93–95%. Seventy-one percent of the scans performed in symptomatic patients showed true-positive findings against 44% in asymptomatic patients. This could have significant implications for the use of salvage radiotherapy (**Figures 3A**–**C**). Stereotactic radiosurgery has been evaluated in several retrospective studies of metastatic gynecological malignancies and has demonstrated activity at various doses and schedules. Particularly in patients with small tumor burden at recurrence and good performance status, the use of stereotactic body radiation therapy (SBRT) to treat FDG-PET avid para-aortic disease has shown a 4-year local control rate of 67.4%, with low incidence of G3-4 complications (Choi et al., 2009; Kunos et al., 2012a,b). More prospective studies are needed to confirm the role of molecular imaging as a routine examination during the follow-up of these patients (Elit et al., 2010).

## RADIOTHERAPY TARGET DEFINITION WITH FDG-PET/CT

The rapid evolution of radiotherapy now makes it possible to deliver HDRs to tumors located near normal structures with explicitly sculpted dose sparing of the normal tissues. Anatomical images have historically been used; however, they lack sensitivity for defining tumor extent, and the capacity to evaluate the biology of the tumor and normal tissue. In this context, the use of anatomical images associated with biological images is essential. Biological images allow mapping of molecular distributions and their surrogates, and can be used to guide external beam radiotherapy. For example, Ma et al. (2011) has shown important tumor volume discrepancies between FDG-PET and MRI probably due to the important geometrical changes in the position of the cervix and corpus uteri as well as variations in bladder and rectal filling. Chan et al. (2008) studied the internal movement of the tumor, cervix, and uterus using weekly cine-MRIs and a point of interest analysis (POI). The fundus POI drifted 1.5 cm caudally during CRT, and the cervical canal 1 cm.

**FIGURE 4:**
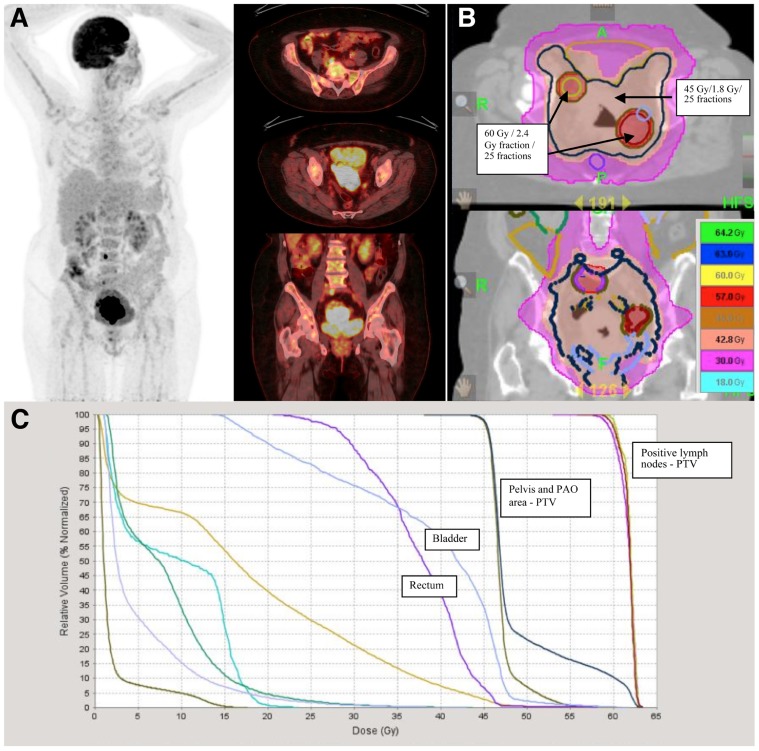
**A 62-year-old lady with FIGO stage IIB cervix cancer and positive pelvic lymph nodes was treated with cisplatin based chemotherapy and radiotherapy**. **(A)** Positive lymph nodes are delineated based on FDG-PET/CT uptake and treated with 60 Gy in 2.4 Gy per fraction in 25 fractions. **(B)** Radiotherapy was delivered with helical tomotherapy. Pelvis and para-aortic areas were treated with 45 Gy in 25 fractions of 1.8 Gy. **(C)** Scale dose banding shows the 95% of the dose. The tumor boost was delivered with MRI-guided brachytherapy in four fractions of 7 Gy.

As previously stated, pathological uptake of FDG-PET may modify treatment strategy, either by extending the radiation volumes to the para-aortic area, or by modifying the dose to the affected lymph nodes (**Figures 4A**–**C**). Esthappan et al. (2004) proposed dose escalation to 59.4 Gy to the positive para-aortic lymph node and 50.4 Gy to the para-aortic region using CT/PET-guided IMRT. In a series of 208 patients with cervix cancer, lymph nodes were scored as either positive or negative for abnormal FDG uptake PET and lymph node status by CT was classified as <1 cm (negative) or >1 cm (positive) (Grigsby et al., 2004). All enlarged lymph nodes detected by CT were PET positive. No patient underwent lymph node dissection. The dose to pelvic lymph nodes was dependent on PET and CT findings: PET negative nodes, <1 cm, 66.8 Gy, and 0/76 failures; PET positive nodes, <1 cm, 66.8 Gy, and 3/89 failures; 1.1 to <2 cm, 66.9 Gy, and 0/21 failures; 2.1 to <3 cm, 69.4 Gy, and 2/15 failures; and 3.1 to <4 cm, 74.1 Gy, and 0/5 failures. The risk of isolated nodal failure was <2%. Nevertheless, most of the patients with para-aortic positive lymph nodes failed at distant sites. The use of higher doses of radiotherapy might only help to increase loco-regional control. For instance the GOG protocol 125 has studied the feasibility of administering chemotherapy and extended field radiotherapy, and showed that in patients with positive para-aortic lymph nodes the combined treatment achieves 33% of progression-free interval at 3 years, this supports the idea that the treatment of para-aortic nodes is important but that better systemic treatments are needed to avoid distant metastasis (Varia et al., 1998).

Not only can FDG-PET/CT drive tumor dose painting with IMRT, but it might also help to limit hematological toxicity. In locally advanced cervical cancer treated with CRT, both modalities are myelosuppressive (Green et al., 2001; Bachtiary et al., 2005; Vale et al., 2010). Identifying active bone marrow sub-regions with FDG-PET might facilitate bone marrow sparing and improve tolerance to chemotherapy (Mell et al., 2006). In a recent study reported by Rose et al. (2012), a strong correlation was observed between radiation dose-volume histogram on the active area of the bone marrow identified by FDG-PET, and the development of hematological toxicity. IMRT can reduce the dose to bone marrow sub-regions identified by FDG-PET/CT: the mean functional bone marrow V10 (volume of bone marrow receiving ≥10 Gy), and V20 (volume receiving ≥20 Gy) has been shown to be significantly less with total bone marrow sparing IMRT (Liang et al., 2012).

This has important implications in the development of new therapeutic strategies to treat cervical cancer. A recently published trial identified a survival advantage in patients with locally advanced cervical cancer treated with concurrent gemcitabine, cisplatin, and pelvic radiation with adjuvant gemcitabine and cisplatin compared with concurrent cisplatin and pelvic radiation alone. In this study, more than Grade-3 hematological toxicity occurred in 72% of the experimental arm and was a frequent cause of treatment discontinuation (Duenas-Gonzalez et al., 2011). Several research groups are now focusing on the implementation of phase III trials looking at the potential benefits of adjuvant chemotherapy (NCT01414608). Consequently, reducing radiation-induced bone marrow damage is essential.

## ROLE OF FDG-PET IN BRACHYTHERAPY

The use of image-guided brachytherapy has become standard in our clinic as well as many other cancer centers. MRI-guided brachytherapy is the method most frequently used, allowing an accurate tumor delineation and dose optimization. Recommendations have been published to avoid inter-observer variability in the delineation of tumors and organs at risk as well as a reliable definition of target volumes with a common language among centers (Haie-Meder et al., 2005; Potter et al., 2006).

A few studies have assessed the role of FDG-PET-guided brachytherapy. Malyapa et al. (2002) Compared two-dimensional (2D) treatment planning orthogonal radiography-based brachytherapy with 3D treatment planning based on FDG-PET in 11 patients with cervical cancer. The patients underwent two PETs: a first one to visualize the tumor and a second one with the FDG placed inside the tandem and ovoid applicators to visualize the treatment source positions for 3D treatment planning. The authors concluded that this technique was feasible and accurate relative to 2D treatment planning. Lin et al. (2007) conducted a dosimetric study comparing intracavitary brachytherapy using a standard plan with a PET-defined tumor volume in 11 patients undergoing intracavitary treatments. The coverage of the target isodose surface for the first implant with and without optimization was 73 and 68%, respectively (*p* = 0.21). For the mid and final implant, the coverage was 83 and 70% (*p* = 0.02). The dose to point A was significantly higher with the optimized plans for both the first implant (*p* = 0.02) and the mid and last implants (*p* = 0.008). The dose to the 2 and 5 cm^3^ of bladder or rectum were not significantly different. The authors concluded that FDG-PET-based treatment planning improved tumor dose coverage without significantly increasing doses to the bladder and rectum. A recent publication by Nam et al. (2012) confirms these results; they evaluated the feasibility of FDG-PET/CT conformal brachytherapy in 12 patients with cervical cancer. Brachytherapy was performed at 41.4 Gy, and the prescribed dose to point A was 4 Gy. The median dose that encompassed 95% of the target volume (D95) of the CTV was 3.23 Gy for point A-2D-based plan vs. 3.99 Gy for the FDG-PET/CT optimized plan. They concluded that PET/CT conformal brachytherapy was feasible and target coverage was better than conventional point A plans.

## ASSESSING TUMOR HYPOXIA BY PET

The most extensively studied biological predictor of response to radiotherapy is hypoxia. Hypoxic cells are more resistant to killing by ionizing radiation and chemotherapy (Brown and Giaccia, 1998).

In general, cervical cancer hypoxia has been associated with more malignant phenotypes (Hockel et al., 1999), higher rates of metastatic disease (Lyng et al., 2000; Fyles et al., 2002, 2006), and higher recurrence rates regardless of whether treatment is RT or surgery (Hockel et al., 1996). Hypoxia coupled with abnormal angiogenesis will provoke impaired tumor perfusion and high interstitial fluid pressure (IFP) which has been further linked with worst outcome (Milosevic et al., 2004).

Several hypoxic tracers suitable for PET have received special attention. Fluoromisonidazole (18-FMISO) is the hypoxia tracer most extensively studied (Rasey et al., 1987, 1989). However, its major disadvantages refer to its slow clearance kinetics and its high lipophilicity. Another PET tracer under study is ^18^F-fluoroazomycin-arabinoside (^18^FAZA). The feasibility of ^18^FAZA was evaluated recently in patients with advanced cervical cancer in a study performed by Schuetz et al. (2010). Fifteen consecutive patients with locally advanced cervical cancer were treated with CRT. ^18^FAZA-PET scans were performed before, during and after external beam therapy and image-guided brachytherapy. Five patients had visually identifiable tumors on ^18^FAZA-PET scans performed prior to therapy, and four patients before brachytherapy. One of five PET positive patients had incomplete remission 3 months after RT, and one had regional recurrence. Four of ten PET negative patients developed distant metastases. The authors concluded that ^18^FAZA-PET imaging is feasible, however, its predictive and prognostic value in cervical cancer remains to be clarified.

One of the most promising agents currently under study is ^60^Cu-labeled diacetyl-bis (N^4^-methylthiosemicarbazone) (^60^Cu-ATSM). In a preliminary study by Dehdashti et al. (2008), 38 women with locally advanced cervical cancer were evaluated before the initiation of definitive CRT. ^60^Cu-ATSM uptake was evaluated semi quantitatively as the tumor-to-muscle activity ratio (T/M). A log-rang test determined that the T/M cut-off uptake value of >3.5 was significantly associated with worst outcome. Higher uptake of ^60^Cu-ATSM has been shown to correlate with other biomarkers of tumor hypoxia such as vascular endothelial growth factor receptor (VEGF), epidermal growth factor receptor (EGFR), cycloxygenase-2, and carbonic anhydrase-IV (Grigsby et al., 2007).

Most clinical copper-ATSM studies have used the agent labeled with the short-lived positron-emitting radionuclide of copper, ^60^Cu (half-life, 0.395 h; β1-decay, 92.5%; electron capture, 7.5%; Dehdashti et al., 2003). To enable copper-ATSM to be translated for use in PET centers that do not have an in-house cyclotron, copper-ATSM labeled with one of the longer-lived positron-emitting nuclides, ^64^Cu (half-life, 12.7 h; β1-decay, 17.4%; β2-decay, 38.5%; electron capture, 43%) or ^61^Cu (half-life, 3.33 h; β1-decay, 62%; electron capture, 38%), is required. The longer half-lives of ^64^Cu and ^61^Cu allow for production at a regional center and distribution to PET facilities in a fashion similar to that for ^18^F-labeled radiopharmaceuticals (Blower et al., 1996).

^64^Cu-labeled diacetyl-di(N(4)-methylthiosemicarbazone) (^64^Cu-ATSM) has also been studied in cervical cancer and comparisons with ^60^Cu-ATSM showed better image quality due to reduced noise. Furthermore the pattern and magnitude of tumor uptake of ^60^Cu-ATSM and ^64^Cu-ATSM were similar (Lewis et al., 2008). A multicentre, prospective, phase II study is currently recruiting patients to define the role of pre-therapy ^64^Cu-ATSM in predicting prognosis and determining the behavior of locally advanced cervical cancer (NCT00794339).

The development of new PET tracers targeting hypoxic response is essential because we are now in the era of rationally designed molecularly targeted therapies combined with CRT, which poses a significant challenge not only in evaluating mixed toxicity profiles but also in the evaluation of tumor response. New molecular targets may work by mechanisms unlikely to cause tumor regression, and there remains an important need to develop biomarkers to provide early evidence of drug activity not only in the tumor but also its vasculature.

## ASSESSING TUMOR ANGIOGENESIS BY PET

Targeting the angiogenic pathway is an increasingly important therapeutic strategy for cervix cancer, and recent phase II studies have shown encouraging results (Townsley et al., 2011; Schefter et al., 2012). The choice of agents and combinations is dependent on understanding the biology of cancer and the availability of anticancer agents and their toxicities. Integrin α_v_β_3_ is up-regulated in both tumor cells and angiogenic endothelial cells, making it an attractive therapeutic target. In recent studies in cervix cancer patients the expression of β_3_ integrins, had a significant prognostic impact on outcome according to univariate and multivariate analyses (Gruber et al., 2005). In another study the expression of α_v_β_6_ in cervix cancer correlated with different clinico-pathological parameters and with worse overall and disease-free survival. Over expression of α_v_β_6_ in cervical squamous carcinomas is an unfavorable prognostic factor. This might reflect an increased capacity of α_v_β_6_-expressing tumor cells to migrate in a fibronectin-rich extra cellular matrix (ECM) and/or to activate TGF-β_1_ at the tumor/stroma interface, both of which processes may contribute to cervical cancer progression (Hazelbag et al., 2007).

Tumor-associated vessels express integrin α_v_β_3_ (Brooks et al., 1994a,b). It is possible that increased expression of integrins α_v_β_3_ and α_v_β_5_ allow angiogenic endothelial cells to bind provisional matrix proteins such as vitronectin, fibrinogen, von willebrand factor, osteopontin and fibronectin that are deposited in the tumor microenvironment. These adhesive interactions could provide survival cues and/or traction for invading endothelial cells. Through genetic deletion, or treatment with integrin antagonists, several additional integrins have been identified as crucial for angiogenesis, including α_1_β_1_, α_2_β_1_, α_4_β_1_, α_5_β_1_, α_6_β_1_, α_9_β_1_, and α_6_β_4_ (Avraamides et al., 2008).

Cilengitide (EMD 121974, manufactured by Merck KGaA, Darmstadt, Germany) is an investigational cyclic arginine–glycine–aspartic acid (RGD) containing pentapeptide sequence that selectively inhibits the α_v_β_3__/__5_ integrins (Dechantsreiter et al., 1999). Cilengitide is the first integrin inhibitor to reach phase III clinical trials in glioblastoma, another highly vascularized cancer (Reardon et al., 2008a,b, 2011; Maurer et al., 2009; Stupp et al., 2010). Cilengitide is now being tested in phase II studies in patients with lung, pancreas, head and neck, and prostate cancer in combination with chemotherapy, radiotherapy, and other molecular targeted agents (Beekman et al., 2006; Friess et al., 2006; Vermorken et al., 2011; Alva et al., 2012).

As a result, better vascular imaging techniques are being developed to monitor responsiveness to treatment. In particular, considerable effort has been expended on characterizing integrin antagonists for their ability to specifically deliver diagnostic agents to tumor cells and associated blood vessels. ^68^Ga-NODAGA-RGD is one of them, composed of one pentacyclic motif (RGDyK) and the ^68^Galium-chelating reagent NODAGA. CycloRGD-NODAGA peptide is labeled with ^68^Ga eluted from a ^68^Ge/^68^Ga generator directly on site (GMP) so as to form the ^68^Ga-NODAGA-RGD that will be administrated to the patient. Dosimetry of ^68^Ga-NODAGA-RGD PET/CT has been extrapolated from mice (Buchegger et al., 2011), and this radiopharmaceutical agent is in clinical use in our institution in a Swissmedic-approved study (NCT01608516). Our group is now evaluating the possibility of embarking on a phase I–II study to evaluate toxicity and efficacy of cilengitide combined with CRT in locally advanced cervical cancer.

## CONCLUSION

There is a high level of evidence that FDG-PET/CT plays an essential role in the primary evaluation of cervical carcinoma, particularly in evaluating lymph nodal status and distant metastases, contributing to precise tumor staging and changes in therapeutic attitudes.

In surgical staged patients the diagnostic performance of FDG-PET/CT has shown a sensitivity of >80%, a specificity of >90% for detecting lymph node metastasis.

Positron emission tomography – computed tomography has gained importance in determining prognosis, assessing treatment response and evaluation of disease recurrence. The use of FDG-PET/CT is important to accurately define radiotherapy volumes, spare active bone marrow from high doses of radiation, and deliver more precise brachytherapy. Despite improved survival with the use of CRT, loco-regional control still constitutes a major problem, and other treatments are necessary to improve effectiveness. Advances in the understanding of the tumor microenvironment such as hypoxia, and angiogenesis, open the window to implement new molecular targeted approaches. Advances in biological images like PET/CT have a tremendous impact on the evaluation of treatment response to new therapeutic strategies.

## Conflict of Interest Statement

The authors declare that the research was conducted in the absence of any commercial or financial relationships that could be construed as a potential conflict of interest.
